# Nationwide Multicenter Study of Advanced Endoscopic Resection and Malignant Risk Model for Gastric Myogenic Tumors (GASTRO Trial)

**DOI:** 10.3390/life16010082

**Published:** 2026-01-05

**Authors:** Chih-Tsung Fan, Tze-Yu Shieh, Wen-Hung Hsu, Hsi-Yuan Chien, Ching-Tai Lee, Ming-Yao Chen, Chung-Ying Lee, Wei-Chen Tai, Sz-Iuan Shiu, I-Ching Cheng, Chen-Shuan Chung

**Affiliations:** 1 Division of Gastroenterology and Hepatology, Department of Internal Medicine, Far Eastern Memorial Hospital, New Taipei City 220, Taiwan; fccjam1214@gmail.com; 2 Division of Gastroenterology, Department of Internal Medicine, Mackay Memorial Hospital, Taipei City 104, Taiwan; range0425@gmail.com; 3 Division of Gastroenterology, Kaohsiung Medical University Chung-Ho Memorial Hospital, Kaohsiung City 807, Taiwan; 4 Division of Gastroenterology and Hepatology, Chung Shan Hospital, Taipei City 106, Taiwan; chienhsiyuan@gmail.com; 5 Department of Endoscopy, E-Da Hospital and I-Shou University, Kaohsiung City 824, Taiwan; 6 Division of Gastroenterology and Hepatology, Department of Internal Medicine, Shuang Ho Hospital, Taipei Medical University, New Taipei City 235, Taiwan; 7 Division of Gastroenterology and Hepatology, Department of Internal Medicine, School of Medicine, College of Medicine, Taipei Medical University, Taipei City 110, Taiwan; 8 Taipei Medical University Research Center for Digestive Medicine, Taipei Medical University, Taipei City 110, Taiwan; 9 Division of Hepatogastroenterology, Department of Internal Medicine, Kaohsiung Chang Gung Memorial Hospital, Kaohsiung City 833, Taiwan; 10 Division of Gastroenterology and Hepatology, Department of Internal Medicine, Taichung Veterans General Hospital, Taichung City 407, Taiwan; 11 Department of Critical Care Medicine, Taichung Veterans General Hospital, Taichung City 407, Taiwan; 12 Department of Internal Medicine, Yang Ming Chiao Tung University, Taipei City 112, Taiwan; 13 Division of Gastroenterology, Department of Medicine, Taiwan Adventist Hospital, Taipei City 105, Taiwan; yingzheng1224@gmail.com; 14 Program A, Department of Electrical Engineering, Yuan Ze University, Taoyuan City 320, Taiwan

**Keywords:** subepithelial lesion, myogenic tumor, endoscopic resection, GIST, EUS

## Abstract

**Background/Objectives:** The prevalence of gastric subepithelial lesions (SELs) is rising. Endoscopic resection (ER) technique provides a minimally invasive alternative to manage gastric SELs. This study aims to evaluate the effectiveness and safety of ER for gastric myogenic tumors, and examine predictors for gastrointestinal stromal tumors (GISTs). **Methods:** The retrospective study was conducted between 2012 and 2024 at nine tertiary centers in Taiwan. We enrolled patients with endoscopic ultrasound (EUS)-documented gastric myogenic tumors managed by endoscopic muscular dissection (EMD), endoscopic subserosal dissection (ESSD), submucosal tunneling endoscopic resection (STER), and endoscopic full-thickness resection (EFTR). Clinical manifestation, endoscopic features, and outcomes were analyzed. **Results:** We enrolled 325 patients with 332 lesions [mean EUS size 14.5 mm, 153 (46.1%) leiomyoma, 152 (45.8%) GISTs, 27 (8.1%) other histology]. ER techniques were 193 (58.1%) EMD, 46 (13.9%) ESSD, 28 (8.4%) STER, and 65 (19.6%) EFTR. Technical success, en bloc, and R0 resection rates were 97.0%, 94.3%, and 88.9%, respectively. Twenty-four (9.0%) procedures were shifted to unintentional EFTR, and 21 (6.3%) patients had complications. No recurrence occurred during mean follow-up period of 921.4 days. Two (0.6%) patients died of non-procedure related reasons. Old age, fundus location, heterogeneous echotexture, and exophytic growth pattern were independent risk factors for GIST (all with *p* < 0.05). Using the above factors, we built a prediction model with sensitivity of 77.0%, specificity of 85.6%, and AUC of 0.8771. **Conclusions:** ER is an efficient and safe management for gastric myogenic tumors. The histological type could be predicted by demographic characteristics and EUS features.

## 1. Introduction

The incidence of gastric subepithelial lesions (SELs) is on the rise, attributed to the widespread use of gastroscopy [1]. Approximately 1.9% of patients who undergo gastroscopy are found to have SELs [2]. A subset of these lesions originates from the muscularis propria (MP) layer, which includes gastrointestinal stromal tumors (GISTs), leiomyomas, and schwannomas. It is imperative to identify SELs with malignant potential, such as GISTs, for timely management. Certain characteristics observed in computed tomography (CT) scans, including location of the lesions, size, presence of necrosis, and enhancement during various phases, may assist in distinguishing GISTs from leiomyomas [3]. Endoscopic ultrasonography (EUS) serves as a superior modality for characterizing the features of SELs; however, it still demonstrates a limited accuracy of 43% in predicting histological diagnoses [4,5]. Notable features of EUS that may aid in differentiating GISTs from leiomyomas include heterogeneity, irregular borders, echogenic foci, cystic (or anechoic) spaces, and the presence of dimpling or ulceration [4,6].

Histological assessment is essential for the definitive diagnosis of SELs, necessitating tissue acquisition for effective risk stratification. Proposed methods for obtaining tissue include mucosal incision-assisted biopsy (MIAB), bite-on-bite stacked biopsy, and EUS-guided fine needle aspiration or biopsy [5]. However, the yield rates of these techniques have proven inadequate, failing to provide the mitotic rate necessary for assessing malignant potential [7]. Furthermore, the incidence of bleeding complications associated with forceps biopsy methods can reach as high as 8%, and the small size of the lesions complicates EUS-guided tissue acquisition [8]. Recent advancements in endoscopic resection (ER) and wound closure techniques, such as endoscopic muscularis dissection (EMD), endoscopic subserosal dissection (ESSD), submucosal tunneling endoscopic resection (STER), and endoscopic full-thickness resection (EFTR), have been developed to excise SELs that extend beyond the submucosal layer, facilitating both definitive histological diagnosis and curative resection [9,10,11]. The clinical guidelines from the American College of Gastroenterology (ACG) and the European Society for Medical Oncology (ESMO) recognize ER as an alternative to surgical intervention for small gastric GISTs [4,12]. Additionally, the European Society of Gastrointestinal Endoscopy (ESGE) guidelines recommend considering ER—specifically STER, EFTR, or endoscopic submucosal excavation (ESE)—as a treatment option when there is a clinical indication or when attempts to obtain a diagnosis for the resection of SELs have failed [13]. The objective of this study was to assess the Gastric Stromal Tumor Resection Outcomes (GASTRO Trial) by different ER techniques and identify EUS predictors for diagnosis of GISTs.

## 2. Materials and Methods

### 2.1. Study Design and Enrollment

This multicenter retrospective study was conducted from January 2012 to April 2024 across nine tertiary-care referral centers in Taiwan. All the expert endoscopists in the nine centers had experience of at least 250 cases in advanced endoscopic resection. In terms of the equipment, the choice of electrosurgical units and endoknife were at the discretion of each endoscopist. The study included patients aged 18 years and older who had EUS-documented SELs originating from the muscularis propria layer of the stomach and who underwent ER. The criteria for ER included an initial lesion size greater than 20 mm, an increase in size during follow-up, the presence of high-risk features (such as heterogeneous echotexture, exophytic growth, and irregular borders) observed on EUS, known malignant histology, symptomatic presentation, and patient preference. Patients were excluded from the study if complete EUS or pathological data were unavailable. The study received approval from the Institutional Review Board of Far Eastern Memorial Hospital (FEMH-109047-E), and the requirement for written informed consent was waived due to the anonymized nature of the data analyzed.

### 2.2. Outcome Measurements

Medical records were reviewed to gather demographic and procedure-related information. The classification of risk of stratification for the GIST was according to modified National Institutes of Health consensus criteria (Table A1 in Appendix A). The primary outcome was defined as technical success rate. Secondary outcomes included en bloc rate, R0 resection rate, procedure time (total procedure time, resection and closure time), hospital stay, rate of shifting to unintentional EFTR, complications, recurrence, and mortality rate. Technical success was defined as the successful removal of the SELs through ER, either as an en bloc or piecemeal procedure. The total procedure time was measured from the initial mucosal incision until complete wound closure. Complications were classified as either intra-procedural or delayed perforation and bleeding. Early bleeding was defined as any bleeding occurring within 48 h after the resection, while delayed bleeding referred to bleeding that happened more than 48 h after the procedure.

### 2.3. Procedures in the ER Techniques

**Figure 1:**
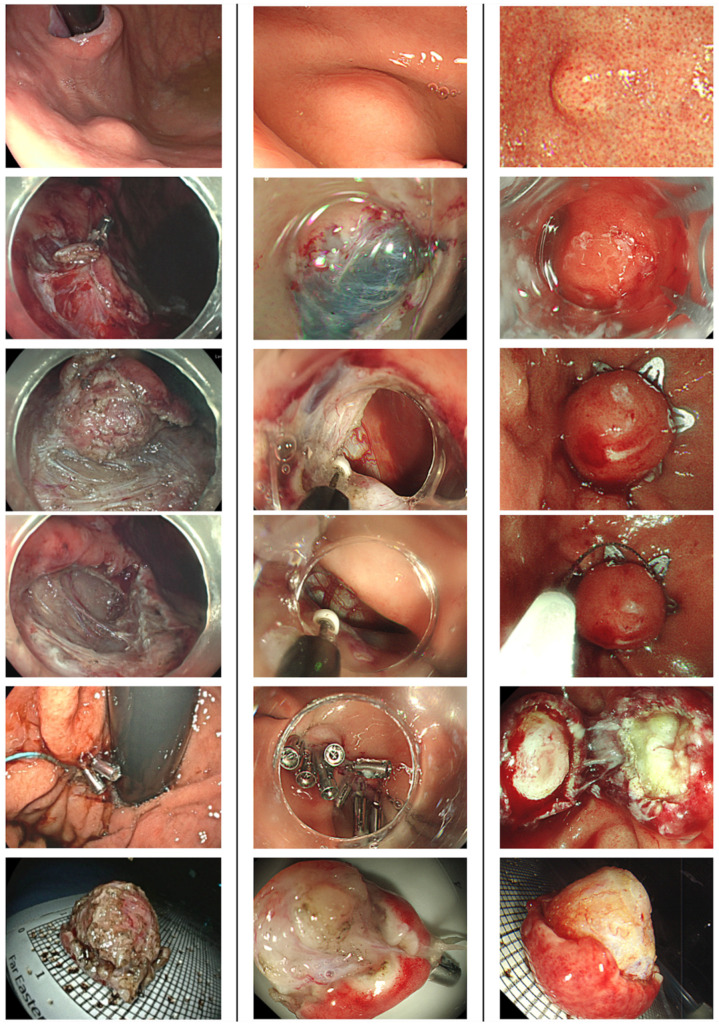
Different endoscopic resection techniques for gastric myogenic tumor. In the figures, we showed the appearance of the lesions before the procedures, during dissection, after closure, and when the lesions were retrieved. (**Left Column**) ESSD. (**Middle Column**) Exposed EFTR. (**Right Column**) Non-exposed EFTR with Padlock clip. ESSD, endoscopic subserosal dissection; EFTR, endoscopic full-thickness resection.

Patients underwent ER under either intravenous or general anesthesia. Preoperative short-term prophylactic antibiotics were administrated [14]. Proton pump inhibitors were prescribed for 8~12 weeks after the procedure. Patients resumed enteral feeding after ER if there were no peritoneal signs noticed. ER technique included EMD, ESSD, STER, and EFTR (Figure 1). The procedural strategies were determined based on the initial evaluation of the lesions, including tumor size and growth pattern (e.g., exophytic versus non-exophytic). EMD and ESSD involved making a mucosal incision, followed by submucosal dissection and muscular or subserosal dissection beneath the visibly discernible tumor margin. EFTR was executed using either a non-exposed method with over-the-scope clips or an exposed method via conventional endoscopic dissection techniques. The choice of traction methods for dissection was left to the discretion of the endoscopists, and was tailored to the growth pattern of the SELs to facilitate en bloc removal. STER was performed by creating a 2 cm mucostomy away from the tumor, followed by submucosal tunneling to locate the SELs, after which tumor dissection, retrieval, and closure of the mucosal entry ensued. All gastric wall defects resulting from ER were securely closed using various techniques, including simple closure with endoclips, purse-string closure with detachable endoloops and endoclips, over-the-scope clips, or an endoscopic suturing system.

### 2.4. Statistical Analysis

The basic characteristics of the patients were evaluated using descriptive statistics. Discrete data were presented as counts and percentages, while continuous variables are expressed as mean values ± standard deviation (SD), along with the maximum and minimum values. To evaluate categorical variables, a chi-squared test was employed. A two-tailed *p* value of less than 0.05 was considered statistically significant. Additionally, logistic regression analysis was performed to assess the relationship between various risk factors and outcomes. Factors associated with histology were identified using univariate and multivariate Cox regression analyses. Variables that yielded *p* values of less than 0.05 in the univariate analysis were included in the multivariate model. Moreover, the receiver operating characteristic (ROC) curve was calculated using the Littenberg and Moses linear model. A points-based risk score was developed based on independent predictors identified from the multivariable logistic regression model. For each independent risk factor, the adjusted odds ratio was transformed using the natural logarithm (β = ln[aOR]). The corresponding β coefficients were then converted into integer risk points by rounding to the nearest whole number. The total risk score for each individual was calculated by summing the points across all risk factors. Risk strata were defined according to the distribution of total scores meaningful cutoffs, and the predicted risk of GIST across score categories was estimated. All statistical analyses were conducted with SPSS for Mac OS, version 29.0.1.0 (IBM Corp., Armonk, NY, USA).

## 3. Results

### 3.1. Demographic Data of Enrolled Patients

**Figure 2:**
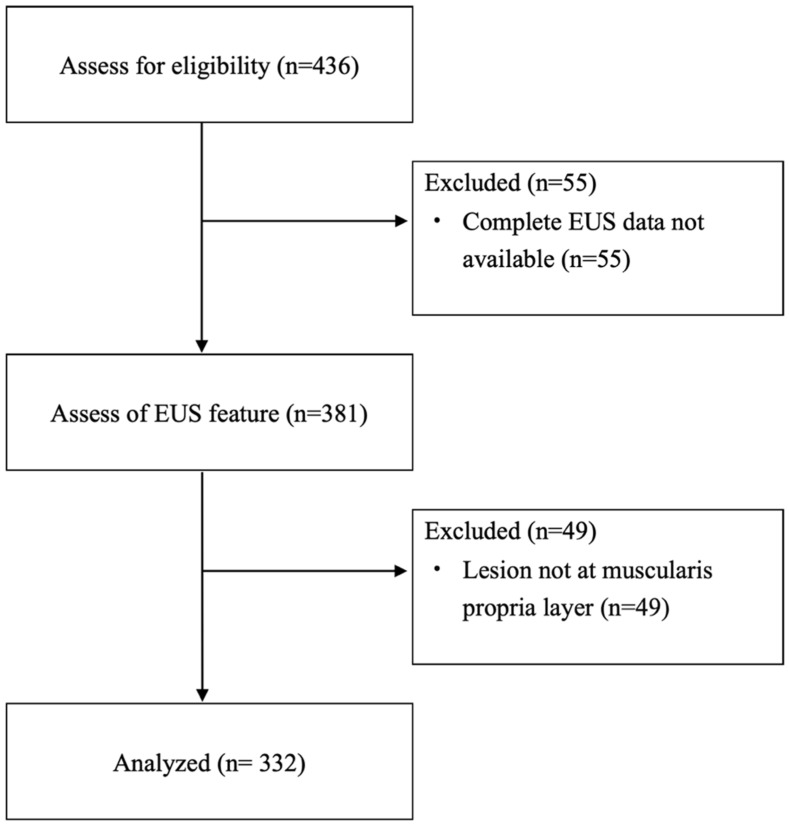
Flowchart of enrollment. EUS, endoscopic ultrasound.

**Figure 3:**
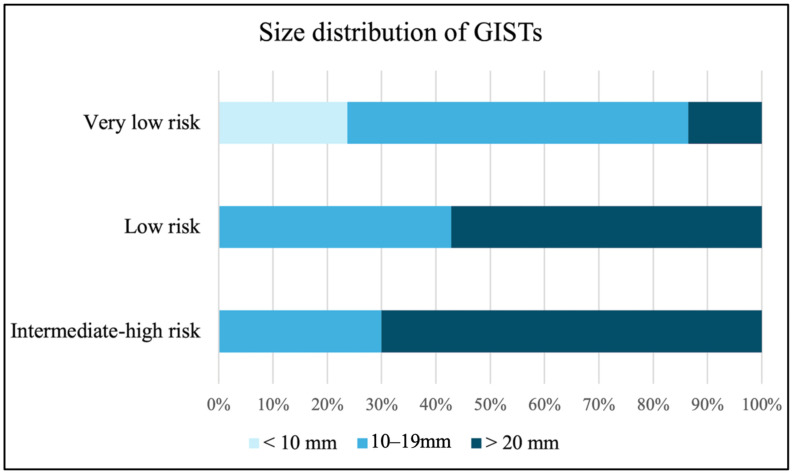
Size distribution of GISTs according to the risk stratification. GIST, gastrointestinal stromal tumor.

The demographic data was presented in Table 1. A total of 325 patients (219 females and 106 males; mean age 55.4 ± 12.5 years) with 332 lesions were enrolled in the study (Figure 2). The mean size (range) ± SD of the myogenic tumors measured under endoscopy was 15.8 (4~50) ± 8.2 mm, while the size under EUS was 14.5 (3~45) ± 7.7 mm. Most lesions were predominantly located in the upper body (41.0%), followed by the cardia (22.9%) and fundus (21.7%). Under EUS examination, the proportions of lesions with irregular borders, heterogeneous echotexture, and exophytic growth were 27.1%, 54.8%, and 33.4%, respectively. The most common pathology identified was leiomyoma (46.1%), followed closely by GISTs at 45.8%. Other histologic types accounted for 8.1% of the tumors and included 8 schwannomas and 5 calcified fibrous tumors. Three lesions were ectopic pancreas and two were spindle cell tumors. The remaining 9 lesions were tubular adenocarcinoma, neuroendocrine tumor, lipoma, ectopic spleen, gastritis cystica polyposa, fundic gland polyp, fibrotic tissue, AV malformation, and one vessel tissue with atherosclerosis. The distribution of pathological diagnosis of each center was listed in Table A2. Among the GISTs, the majority (77.6%) were classified as very low risk, while the remaining tumors were categorized as low risk (9.2%), intermediate risk (9.9%), and high risk (3.3%). Figure 3 illustrates the size distribution of GISTs according to risk stratification, showing that tumors larger than 20 mm were found in 13.6% of very low-risk, 57.1% of low-risk, and 70.0% of intermediate- to high-risk lesions.

### 3.2. Efficacy and Safety of ER in Myogenic SELs

The technical success rate was 97.0%, and the en bloc rate was 94.3% across the 332 procedures, which included EMD, ESSD, STER, and EFTR performed in 58.1%, 13.9%, 8.4%, and 19.6% of cases, respectively. A total of 24 procedures (9.0%) were unintentionally shifted to EFTR. The mean ± SD procedure time was 60.6 ± 44.5 min, which included a resection time of 45.3 ± 39.2 min and a closure time of 11.7 ± 11.0 min (Table 2).

The R0 resection rate was 88.9%. Among the 28 patients who had R1 resection, 25 patients (89.3%) were with GISTs. Of these, 6 patients (24.0%) underwent surgical intervention, and 1 patient (4.0%) received adjuvant chemotherapy due to a high mitotic count risk. The remaining 18 patients (72.0%) were monitored with follow-up alone, which included endoscopy and CT scans every 6 to 12 months for the first 3 years, followed by annual surveillance for 5 years. Notably, none of the patients with R1 resection experienced recurrence during the follow-up period (Table A3).

The mean ± SD hospital stay was 5.0 ± 2.9 days. A total of 21 complications (6.3%) were recorded, with intra-procedural inadvertent perforation being the most common (15 patients, 4.5%), followed by delayed bleeding (2 patients, 0.6%). In total, 16 patients (4.8%) required surgery following the primary procedure. The complication rates for SELs smaller and larger than 20 mm were 3.8% and 17.7%, respectively (*p* = 0.001). Tumors located at proximal stomach (cardia, fundus, and upper body) and larger tumor sizes were found to lead to higher risks of complication (Table A4). Regarding mortality, one 81-year-old patient with a 2.8 cm GIST experienced microperforation with bleeding after ESSD and subsequently underwent surgery. Unfortunately, the patient passed away due to pneumonia and acute respiratory distress syndrome one month later. Another patient with a 3.0 cm GIST died from underlying lung cancer after 5 years of follow-up. No recurrence was observed during the mean follow-up period of 921.4 ± 947.5 days.

### 3.3. Comparison Between Leiomyoma and GIST

We compared demographic data and outcomes between patients with GISTs and leiomyomas. In the univariate analysis, significant differences were observed in EUS features (irregular border, heterogeneous echotexture, and exophytic growth), sex, age, tumor located at fundus and EUS tumor size between the two groups (Table 3). As for the outcomes, ER for GISTs had longer procedure time (71.3 ± 47.7 vs. 46.9 ± 37.1 min, *p* < 0.001), a lower proportion of R0 resection (90.4% vs. 98%, *p* < 0.001), higher risks of complication (7.9% vs. 2%, *p* = 0.027), and unintentional EFTR (12.5% vs. 0%) (Table 3). We also analyzed predictors for higher-risk groups within GISTs. We compared demographic factors between very low-risk GISTs and higher grade (low, intermediate, and high) of GISTs. Larger size in both EUS (OR = 1.160, *p* < 0.001) and endoscopic (OR = 1.161, *p* < 0.001) size are related to higher grades of GISTs. EUS characteristics were not significantly associated with the grading of GISTs (Table A4).

**Figure A1:**
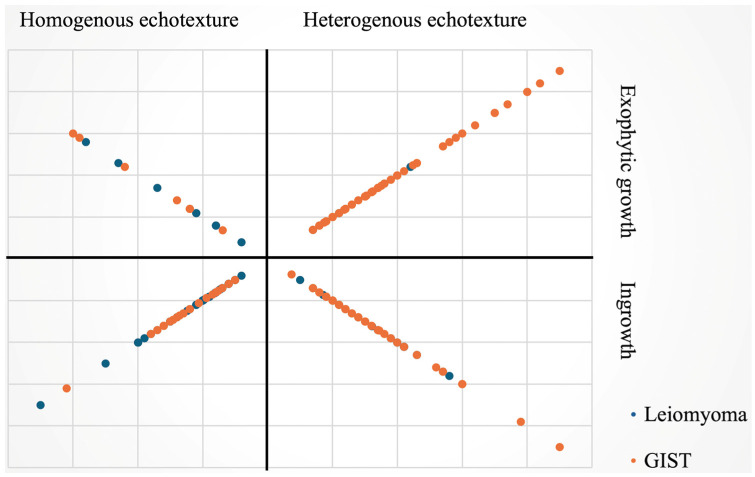
Distribution of EUS size, echotexture, and growth feature with histology. The larger size of the tumors is represented by the farther distance of the dots from the origin. EUS, endoscopic ultrasound; GIST, gastrointestinal stromal tumor.

**Figure A2:**
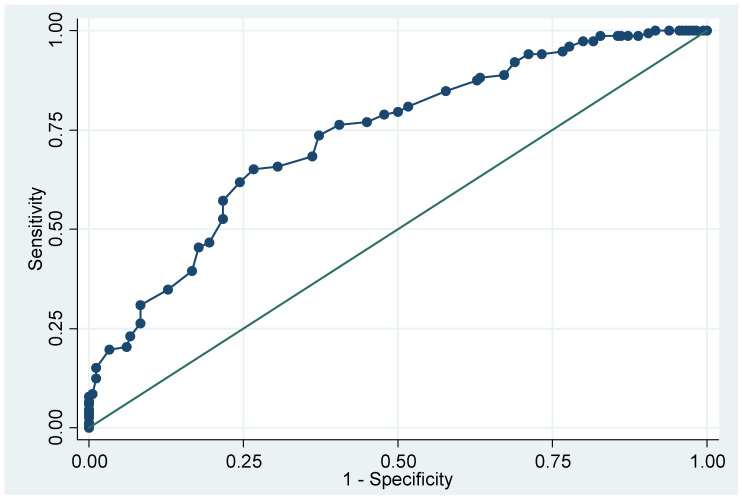
The ROC curve of age for prediction of GIST, AUC 0.7329. ROC, receiver-operation characteristic; GIST, gastrointestinal stromal tumor; AUC, area under the curve.

**Figure A3:**
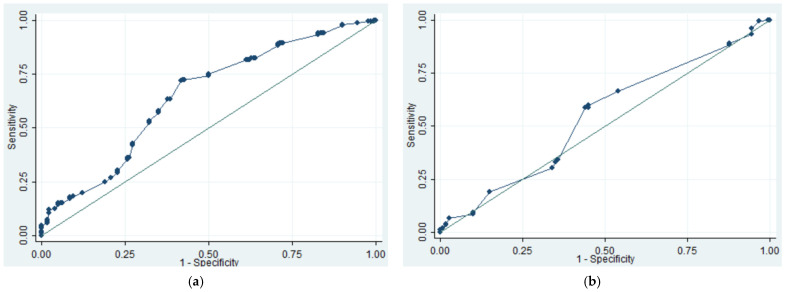
The ROC curves of tumor size for prediction of GIST. (**a**) ROC curve of EUS tumor size. AUC 0.6471. (**b**) ROC curve of endoscopic tumor size. AUC 0.5444. ROC, receiver-operation characteristic; EUS, endoscopic ultrasound; GIST, gastrointestinal stromal tumor; AUC, area under the curve.

The distribution of EUS size and features is illustrated in Figure A1. We also analyzed the threshold for continuous variable (tumor size and age) in prediction of GISTs. When using age 72 as the cutoff level, the sensitivity, specificity, and area under the ROC curve (AUC) were 15.1%, 98.9%, and 0.7329, respectively. For EUS size, a threshold of 12 mm resulted in a sensitivity of 71.7%, specificity of 58.3%, and AUC of 0.6471. In comparison, using an endoscopic size threshold of 15 mm yielded sensitivity of 58.6%, specificity of 56.1%, and AUC of 0.5444 (Figure A2 and Figure A3).

**Figure 4:**
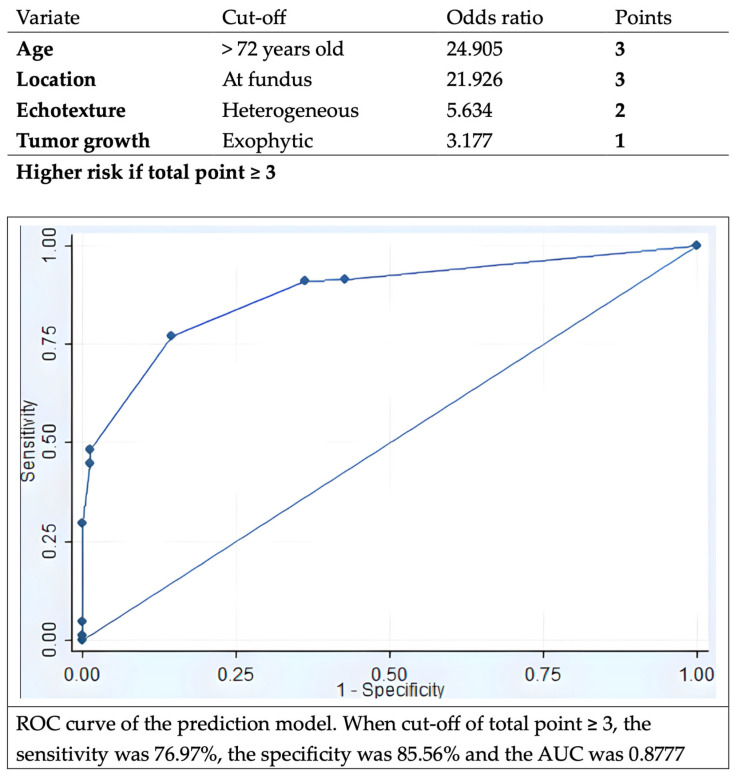
Prediction model for GIST, and the ROC curve of the prediction model with AUC 0.8771. ROC, receiver-operation characteristic; GIST, gastrointestinal stromal tumor; AUC, area under the curve.

The multivariate analysis revealed that GISTs were more prevalent in elderly patients, fundus location, exhibiting heterogeneous echotexture and exophytic growth under EUS (Table 4). Using the adjusted value of logarithm of odds ratio based on the multivariate analysis, we built up a model to predict the malignant potential of the SELs. This model could achieve sensitivity of 77.0%, specificity of 85.6%, and AUC of 0.8777 using threshold of total score 3 (Figure 4).

## 4. Discussion

This retrospective study demonstrates ER being a valid and reliable strategy for the management of gastric SELs originating from MP layer, with high technical success and en bloc rates as well as low complication rate. In addition, EUS features, including heterogeneous echotexture and exophytic growth rather than the tumor size, could be used to predict malignant potential of gastric myogenic tumors. A model with factors of age, location at fundus, and EUS features aid in predicting malignant potential of gastric myogenic tumors.

The prevalence of GISTs is estimated to be 10 to 15 per 100,000 in the general population and counts for up to 3% for malignancies in the GI tract [15]. The incidence of GISTs was reported to be as high as up to 2 per 100,000 in some studies [16]. The natural course of gastric SELs is still poorly understood; yet, about 8.4% of the lesions were found with enlargement during serial endoscopy follow-up [2]. Proportions with malignant potential within gastric SELs were reported in some studies, at around 23–34% [17]. One cohort revealed that 35.1% small (i.e., less than 2 cm) gastric SELs were GISTs [18]. In updated international guidelines, the malignant potential of GISTs was emphasized [12,19]. The World Health Organization (WHO) classified GIST as a malignant tumor, disregarding location, tumor size, and mitotic count [20]. Within our cases, almost half (45.8%) of gastric SELs originating from the MP layer are GISTs, even those of smaller sizes. Resection of all gastric myogenic tumors disregarding tumor size, particularly in those with high-risk EUS features, should be taken into consideration as the appropriate initial management.

Resection is recommended as standard treatment for GISTs larger than 20 mm [4,19]. Based on recent evidence, the ESGE guideline suggests that surveillance or resection are both acceptable in small (<20 mm) proven GISTs in the stomach [13]. Nevertheless, ESMO guidelines suggest complete excision of all GISTs regardless of size, and ER could be considered for small tumors to minimize morbidity [12]. The American College of Gastroenterology (ACG) and ESGE guidelines also describe ER as alternative therapies in gastric GISTs < 20 mm to avoid long-term surveillance [4,13]. More evidence supports ER in managing GISTs, due to shorter procedure times, better post operative recovery without increased of recurrence or complications compared with operation [21]. Nevertheless, a higher proportion of R1 resection in ER compared to surgery was observed, although this phenomenon was not related to a higher recurrence rate [22,23]. In our study, the R0 resection rate is slightly lower than 90%, although there was no recurrence noted during follow-up. We believe that the electrocauterization artifacts contributed to the pathological findings of R1 resection. As a result, there was no recurrence after such an R1 resection rate. Another possible reason for low recurrence was that the predominance of lesions was for very low-risk GISTs in this study, which are characterized by slow growth rate. Complication rate for ER in GISTs was disclosed between 0% and 14.4% [24,25]. Pneumoperitoneum was described at about 10.6% in a large-scale study of EFTR [25]. However, perforation may not be seen as a severe adverse event if successful closure is completed, especially in EFTR with intentional perforating gastric wall. Therefore, the true “complication” is hard to define. The complication rate in our cohort is 6.3% while inadvertent perforation is the most common one. About one-third of patients with perforation needed an additional operation while another two-thirds of patients underwent either intra-procedure needle decompression with successful endoscopic closure and conservative treatment with antibiotics. Two mortalities were recorded during follow-up but none of them were related to the procedure or the tumor.

Advancements in techniques of ER beyond the submucosal layer provide alternative minimally invasive treatment options for GISTs. EMD was found to have a complete resection rate of 96%, with some chance of perforation able to be managed by endoscopic techniques [9]. ESSD was reported to have a similar complete resection, en bloc rate, and adverse event with EMD [10,26]. The efficacy and safety of STER and EFTR for the management of gastric myogenic tumors were also proven [27]. Either exposed or non-exposed EFTR were found to have technical success rates close to 100% without major adverse events [11]. One analysis comparing EFTR with STER showed earlier enteric feeding and shorter stay after STER, with a higher en bloc rate in EFTR [28]. This may be due to the chance of breaching the tumor capsule when dissection in tunnel is accomplished by STER. Total complication rate was 6.5% in the analysis and there was no difference between these two procedures [25]. Our study demonstrates high success and en bloc rate using variant types of ER procedures. Amount 24 procedures were classified as unintentional EFTR, nineteen of them were GISTs. This may be because of larger size and exophytic growth of GISTs. For the diagnostic value of image study, one recent study found that artificial intelligence-based models using CT features demonstrated good performance for prediction of risk stratification of GISTs [29]. According to our analysis, the EUS evaluation, heterogeneous echotexture, and exophytic growth under EUS may indicate higher risk of SELs with malignancy potential. EUS features for prediction of GISTs were conducted in multiple studies. A cohort found heterogeneity and marginal halo signs were more observed in GISTs while irregular border as well as cystic change under EUS did not reach a significant difference [30]. This is similar to our result that heterogeneous echotexture was more important than irregular border. Another study with 138 patients with larger (mean size 34 mm) SELs revealed that non-smooth border, blurring of the layers, presence of blood flow or special inner structure, hypoechoic and heterogeneous echotexture were related to histology of GISTs [31]. As for correlation of EUS findings with risk stratification, one study suggested EUS features are not reliable for prediction and that only tumor size is related to mitotic count [32]. The findings of our study were comparable to previous studies. Using ROC analysis, we found 15 mm for EUS size and 12 mm for endoscopic size were cut point for higher risk being SELs with malignant potential. Nevertheless, low AUC values were found in both groups. One study enrolled small SELs for suspected GIST and found that lesions of more than 9.5 mm had a significantly higher risk for tumor growth [33]. The size necessary to predict malignant potential of gastric myogenic tumor is still debatable. One study developed a scoring system using CT and EUS features to differentiate GIST and schwannoma from leiomyoma, for which tumor location and uneven echogenicity were also suggested as key factors [34]. As for prediction of GIST, one retrospective study used multiple variants of demographic data, and EUS features were used to distinguish GIST from leiomyoma, showing a high AUC [6]. Compared to the prediction model we built, ours might be more simple to apply clinically. We apply only four significant factors (aged over 72, fundus location, heterogeneous echotexture, and exophytic growing), and do not include tumor size. This means that even small lesions could be evaluated by this model. Additionally, we simplified the points of each variant, making it easy to calculate the score. We believe that this prediction model may aid clinicians in deciding whether endoscopic resection should be considered directly or after failed multiple attempts of tissue acquisition, as well as predicting a higher risk of complications and inadvertently perforation, enabling the preparation of suitable closure devices in advance.

There are some limitations of this study. First, this is a multicenter, retrospective study. Therefore, heterogenicity in determination of endoscopic procedures, including resection methods as well as closure techniques may occur. Second, the EUS images are evaluated by endoscopists at each study institute rather than from central reading. Interobserver variations should be considered. Third, the prediction model was only internal validated at present. For clinical practice, endoscopists still need to evaluate the lesion, respectively, upon their characteristics, including the factors that were not mentioned in our prediction model. Furthermore, a future prospective study is therefore warranted to achieve external validation of the model. Lastly, there is wide range of follow-up periods of the patients. Recurrence in those patients with R1 resection of GISTs with lower risk may not be discernible during the study period.

## 5. Conclusions

In conclusion, ER appears to be an efficient and safe method for precise histological assessment and for management of gastric myogenic tumors. Additionally, the malignant potential of gastric myogenic tumors could be predicted by examining EUS features as well as demographic characteristics. More research on long-term data about the outcomes of patients with gastric myogenic tumors managed by ER is warranted.

## Figures and Tables

**Table 1:** Demographic data of enrolled subjects.

Variables	Results
Total patients	325
Total lesions	332
Sex, number (%)	
Female	219 (66.0)
Male	106 (34.0)
Age, mean ± SD (range), years old	55.4 ± 12.5 (22~87)
Tumor location, number (%)	
Fundus	72 (21.7)
Cardia	76 (22.9)
Upper body	136 (41.0)
Middle body	24 (7.2)
Lower body	17 (5.1)
Antrum	6 (1.8)
Angularis	1 (0.3)
Endoscopic tumor size, mean ± SD (range), mm	15.8 ± 8.2 (4–50)
EUS tumor size, mean ± SD (range), mm	14.5 ± 7.7 (3–45)
EUS characteristic, number (%)	
Border	
Regular	242 (72.9)
Irregular	90 (27.1)
Echotexture	
Homogeneous	150 (45.2)
Heterogeneous	182 (54.8)
Growth	
Ingrowth	221 (66.6)
Exophytic	111 (33.4)
Histology type, number (%)	
Leiomyoma	153 (46.1)
GIST	152 (45.8)
Very lower risk	118 (77.6)
Low risk	14 (9.2)
Intermediate risk	15 (9.9)
High risk	5 (3.3)
Other	27 (8.1)

SD, standard deviation; EUS, endoscopic ultrasound; GIST, gastrointestinal stromal tumor.

**Table 2:** Procedural related outcomes and follow-up data.

Variables	Results
Intention procedure	
EMD, number (%)	193 (58.1)
ESSD, number (%)	46 (13.9)
STER, number (%)	28 (8.4)
EFTR, number (%)	65 (19.6)
Technical success, number (%)	322 (97.0)
En bloc resection, number (%)	313 (94.3)
R0 resection, number (%)	295 (88.9)
Procedure time, mean ± SD (range), minute	60.6 ± 44.5 (3~315)
Resection time, mean ± SD (range), minute	45.3 ± 39.2 (2~300)
Closure time, mean ± SD (range), minute	11.7 ± 11.0 (1~80)
Unintentional shift to EFTR, number (%)	24 (9.0)
Hospital stays, mean ± SD (range), day	5.0 ± 2.9 (1~40)
Complication, number (%)	21 (6.3)
Early bleeding (<48 h), number (%)	0 (0.0)
Delay bleeding (>48 h), number (%)	2 (0.6)
Intra-procedural inadvertent perforation, number (%)	15 (4.5)
Delay perforation, number (%)	1 (0.3)
Other *, number (%)	3 (0.9)
Additional therapy following the procedure, number (%)	17 (5.1)
Surgical resection, number (%)	16 (4.8)
Chemotherapy, number (%)	1 (0.3)
Endoscopic resection, number (%)	0 (0.0)
Mortality †, number (%)	2 (0.6)
Recurrence, number (%)	0 (0.0)
Follow-up period, mean ± SD (range), day	921.4 ± 947.5 (7–4172)

EMD, endoscopic muscularis dissection; ESSD, endoscopic subserosal dissection; STER, submucosal tunneling endoscopic resection; EFTR, endoscopic full-thickness resection; SD, standard deviation. * One patient with gastric wall abscess, another had post-anesthesia vomiting. The other patient was recorded to have blood-tingled sputum and mild epistaxis. † Two patients died after the procedure. One patient expired due to pneumonia and acute respiratory distress syndrome 1 month after the procedure. Another patient expired due to underlying lung cancer after 5 years of follow-up.

**Table 3:** Comparison between leiomyoma and GIST.

	Leiomyoma (*n* = 153)	GIST (*n* = 152)	*p* Value
Male sex, number (%)	35 (22.9)	59 (38.8)	0.003
Age, mean ± SD (range), years old	50.6 ± 12.0 (23–73)	61.1 ± 10.4 (33–87)	<0.001
Endoscopic tumor size, mean ± SD (range), mm	14.9 ± 7.8 (4–45)	16.4 ± 8.6 (5–50)	0.099
EUS tumor size, mean ± SD (range), mm	12.0 ± 5.7 (4–35)	16.5 ± 8.4 (3.7–45)	<0.001
<10 mm, number (%)	66 (43.1)	28 (18.4)	
10~19 mm, number (%)	64 (41.8)	86 (56.5)	
>20 mm, number (%)	23 (15.0)	38 (25.0)	
EUS characteristic, number (%)			
Irregular border	27 (17.6)	57 (37.5)	<0.001
Heterogeneous echotexture	46 (30.1)	122 (80.3)	<0.001
Exophytic growth	20 (13.1)	80 (52.6)	<0.001
Located at fundus (%)	4 (2.6)	67 (44.1)	<0.001
Procedure time, mean ± SD (range), minute	46.9 ± 37.1 (3–315)	71.3 ± 47.7 (4–291)	<0.001
Technical success, number (%)	152 (99.3)	146 (96.1)	0.092
En bloc resection, number (%)	147 (96.7)	142 (97.3)	0.782
R0 resection, number (%)	149 (98.0)	132 (90.4)	<0.001
Unintentional shift to EFTR, number (%)	0 (0.0)	19 (12.5)	NA
Complication, number (%)	3 (2.0)	12 (7.9)	0.027

GIST, gastrointestinal stromal tumor; SD, standard deviation; EUS, endoscopic ultrasound; EFTR, Endoscopic full-thickness resection; NA, not applicable.

**Table 4:** Predictors for histological GIST of gastric SELs *.

	Univariate		Multivariate	
	OR (95% CI)	*p* Value	OR (95% CI)	*p* Value
Male sex	1.795 (1.127–2.861)	0.014	1.870 (0.980–3.568)	0.057
Age > 72 years old	15.868 (3.676–68.504)	<0.001	24.905 (4.822–128.638)	<0.001
EUS tumor size	1.071 (1.037–1.106)	<0.001	1.008 (0.966–1.051)	0.709
Located at fundus	27.588 (10.724–70.970)	<0.001	21.926 (7.932–60.609)	<0.001
EUS characteristic				
Irregular border	2.673 (1.620–4.408)	<0.001	1.075 (0.547–2.775)	0.834
Heterogeneous echotexture	8.133 (4.906–13.484)	<0.001	5.634 (2.843–11.162)	<0.001
Exophytic growth	5.341 (3.326–8.814)	<0.001	3.177 (1.664–6.069)	<0.001

GIST, gastrointestinal stromal tumor; SEL, subepithelial lesion; EUS, endoscopic ultrasound; CI, confidence interval. * GISTs, leiomyoma, and tumors with other histology are all included in this analysis.

## Data Availability

The datasets generated during the current study are available from the corresponding author on reasonable request.

## Abbreviations

ACG	American College of Gastroenterology
AUC	Area under the receiver operating characteristic curve
CT	Computed tomography
EFTR	Endoscopic full-thickness resection
EMD	Endoscopic muscular dissection
ER	Endoscopic resection
ESE	Endoscopic submucosal excavation
ESGE	European Society of Gastrointestinal Endoscopy
ESMO	European Society for Medical Oncology
ESSD	Endoscopic subserosal dissection
EUS	Endoscopic ultrasound
GIST	Gastrointestinal stromal tumor
HPF	High-power field
MIAB	Mucosal incision-assisted biopsy
MP	Muscularis propria
ROC	Receiver operating characteristic
SD	Standard deviation
SEL	Subepithelial lesions
STER	Submucosal tunneling endoscopic resection
WHO	World Health Organization

## Appendix A

**Table A1:** Risk stratification of GIST according to the National Institutes of Health consensus criteria.

Risk Category	Tumor Size	Mitotic Count (per 50 HPF)
Very low risk	<2	<5
Low risk	2–5	<5
Intermediate risk	<5	6–10
	5–10	<5
High risk	>5	>5
	>10	Any
	Any	>10

HPF, High-power field.

**Table A2:** List of distribution of pathological diagnosis for each center. The centers were arranged in order of total lesion numbers.

Centers	Total Lesion Number	GIST	Leiomyoma	SEL of Other Types of Histology
Center 1	155	77	69	9
Center 2	72	33	32	7
Center 3	35	14	20	1
Center 4	16	5	11	0
Center 5	15	6	7	2
Center 6	14	7	5	2
Center 7	11	3	4	4
Center 8	10	7	3	0
Center 9	4	0	2	2
Total	332	152	153	27

GIST, gastrointestinal stromal tumor. SEL, subepithelial lesion.

**Table A3:** Lists of additional therapies among patients with GIST who had R1 resection.

Risk Stratification of GIST	Additional Therapy	Number
Very low risk	No additional therapy	14
	Operation	4
Low risk	No additional therapy	4
High risk	Operation	2
	Chemotherapy	1

GIST, gastrointestinal stromal tumor.

**Table A4:** Risk factors of complication during ER.

	OR (95% CI)	*p* Value
EUS tumor size	1.052 (1.004–1.103)	0.035
Tumor located at proximal stomach *	4.292 (1.672–11.015)	0.002
Histology of GIST	1.629 (0.667–3.976)	0.284

* The proximal stomach here included cardia, fundus and upper body. ER, endoscopic resection; GIST, gastrointestinal stromal tumor.

**Table A5:** Analyses of demographic data for prediction of staging of GIST: “Very low risk GISTs” versus “Not very low risk GISTs” *.

	OR (95% CI)	*p* Value
Male sex	0.693 (0.311–1.563)	0.381
Age, years old	1.023 (0.985–1.063)	0.233
Endoscopic tumor size, mm	1.161 (1.094–1.232)	<0.001
EUS tumor size, mm	1.160 (1.096–1.227)	<0.001
EUS characteristic		
Irregular border	0.749 (0.334–1.680)	0.482
Heterogeneous echotexture	1.191 (0.443–3.204)	0.728
Exophytic growth	1.016 (0.473–2.182)	0.967

GIST, gastrointestinal stromal tumor; CI, confidence interval. * Not very low risk of GISTs including low-, intermediate-, and high-risk groups.
